# Reshaping Treatment of Heart Failure with Preserved Ejection Fraction

**DOI:** 10.3390/jcm11133706

**Published:** 2022-06-27

**Authors:** Nikolaos Karamichalakis, Andrew Xanthopoulos, Filippos Triposkiadis, Ioannis Paraskevaidis, Elias Tsougos

**Affiliations:** 1 6th Department of Cardiology, Hygeia Hospital, 15123 Athens, Greece; karamichalakisnick@outlook.com (N.K.); iparas@otenet.gr (I.P.); tsougos@yahoo.com (E.T.); 2 Department of Cardiology, University Hospital of Larissa, 41110 Larissa, Greece; andrewvxanth@gmail.com

**Keywords:** heart failure, preserved, left ventricular ejection fraction, neurohormonal inhibitors, sodium-glucose cotransporter 2 inhibitors, treatment

## Abstract

Current data indicate that in the community, approximately 50% of patients with heart failure (HF) have preserved left ventricular (LV) ejection fraction (LVEF)—the so-called HFpEF. Treatment of HFpEF has been considered an unmet need for decades. We believe that the main underlying reasons have been (a) the ever-changing LVEF cut-offs used for HF classification; (b) controversies regarding the definition of the LVEF normal range; (c) the fact that HFpEF does not represent a phenotype, but a category of diseases with entirely different characteristics (hypertensive heart disease, valvular heart disease (VHD), hypertrophic cardiomyopathy (HCM) etc.); (d) the lack of recognition that hypertensive HFpEF is the most common and important HFpEF phenotype; (e) the assumption that neurohormonal overactivity is absent in HF patients with a LVEF > 45–50% which has been proven to be wrong. Current HFpEF trials, in which the vast majority of the participants suffered from hypertension (HTN), whereas VHD and HCM were absent, demonstrated that neurohormonal and sodium-glucose cotransporter 2 (SGLT2) inhibitors are effective in HF patients over a wide LVEF range. Thus, restricting these lifesaving treatments to HF patients with reduced LVEF is not justified anymore and it should be additionally considered for HFpEF patients suffering from HTN.

## 1. Introduction

The clinical syndrome of heart failure (HF) remains a worrying healthcare issue, as it affects more than 26 million people worldwide, despite the current drug and device therapies [1,2]. In the community, approximately 50% of patients with HF suffer from HFpEF (HF with preserved left ventricular (LV) ejection fraction (LVEF) and although the age-specific incidence of HF is decreasing, this trend is less dramatic for HFpEF than for HF with reduced LVEF (HFrEF) [3]. The lack of effective treatments for HFpEF has been attributed to several reasons, including the absence of animal models that accurately recapitulate the complexities of the human disease [4]. In this paper we argue that there are treatments that are effective in most HFpEF patients, especially those in whom hypertension (HTN) is present, provided that the severe limitations of the arbitrary LVEF cut-offs used for HF classification are recognized and the contribution of HTN to HFpEF pathogenesis is given the credit that it deserves.

## 2. LVEF in Heart Failure Classification and Treatment Guidance

LVEF has been used for decades for HF classification and treatment guidance. The 2013 American College of Cardiology Foundation (ACCF)/American Heart Association (AHA) guidelines defined HFrEF by a LVEF ≤ 40%, borderline HFpEF by a LVEF 41–49%, and HFpEF by a LVEF ≥ 50% [5]. In contrast, the National Heart Foundation of Australia and the Cardiac Society of Australia and New Zealand guidelines defined HFrEF and HFpEF by a LEV < 50% and ≥ 50%, respectively, and did not recognize borderline HFpEF or HF with midrange or mildly reduced LVEF (HFmrEF) as a distinct entity [6]. Further, in the recent Universal Definition and Classification of Heart Failure [7], which was adopted by the European Society of Cardiology [8], HF classification includes HFrEF with an LVEF of ≤ 40%, HFmrEF with an LVEF of 41–49%, and HFpEF with a LVEF of ≥50%. Shortly after, another classification of HF was proposed, which defined HFrEF by a LVEF < 40%, HFmrEF by 40% ≤ LVEF < normal, and “heart failure with normal LVEF” (hFnEF) by a LVEF of ≥55% in men and ≥60% in women [9]. Another term introduced in the 2022 ACCF/AHA Guideline for the Management of Heart Failure, is HF with improved LVEF (HFimpEF) to characterize a specific subgroup of the hFrEF population [10]. This confusion regarding the LVEF cut offs has had a dramatic impact, especially at the higher end of the HF spectrum, as the poorly defined HFpEF is not a phenotype but a category of diseases with entirely different characteristics whose prevalence significantly varies from study to study. For example, in the Irbesartan in Heart Failure with Preserved Ejection Fraction Study (I-PRESERVE) the vast majority of participants (approximately 90%) suffered from HTN and patients with valvular heart disease (VHD) or hypertrophic cardiomyopathy (HCM) were virtually absent [11], whereas in some trials demonstrating phenotypic persistence (no change in LVEF) in HFpEF approximately half of the participants suffered from VHD and HCM [12].

A prerequisite for using the terms for mildly reduced, preserved, or normal LVEF is a definition of the normal LVEF range. According to the 2015 recommendations of the American Society of Echocardiography and the European Association of Cardiovascular Imaging, the normal reference range for LVEF is 52–72% for males and 54–74% for females [13]. The latest guidelines from the British Society of Echocardiography define as normal (preserved) a LVEF ≥ 55% [14]. However, several recent studies raise serious concerns regarding the normal LVEF ranges proposed by the echocardiographic societies.

**Figure 1:**
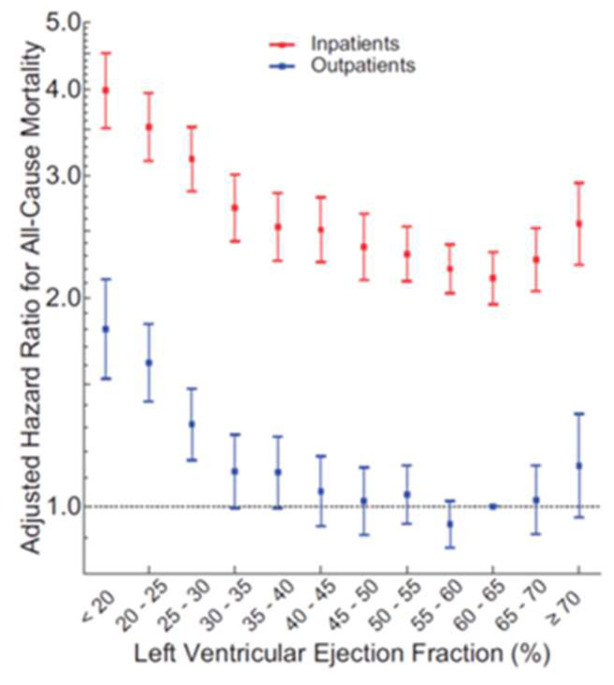
Left ventricular ejection fraction (LVEF) adjusted hazard ratios (HR) in patients with heart failure. Reprinted with permission from Wehner GJ, Jing L, Haggerty CM et al. Routinely reported ejection fraction and mortality in clinical practice: where does the nadir of risk lie? Eur. Heart J. 2020;41:1249–1257 [15]. Copyright © The Author(s) 2019. Published on behalf of the European Society of Cardiology. This article is published and distributed under the terms of the Oxford University Press, Standard Journals Publication Model.

Gladding et al., investigated the relationship between echocardiographically obtained LVEF and survival and observed that during follow-up the unadjusted hazard ratios (HR) for mortality demonstrated a U-shaped relationship for LVEF with a nadir of risk at an LVEF of 60–65%, with the results being similar after adjustments for conditions accompanied by an elevated LVEF (mitral regurgitation, increased wall thickness, and anemia) and when restricted to patients suffering from HF (Figure 1) [15].

**Figure 2:**
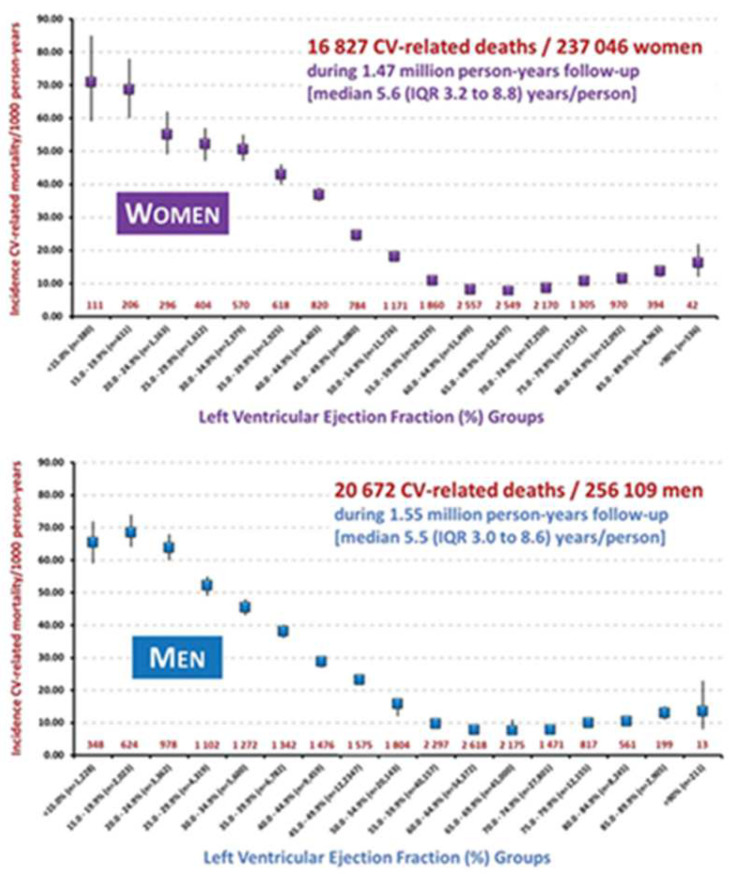
Sex-related differences in the incidence rate of cardiovascular (CV) mortality. Reprinted with permission from Stewart S, Playford D, Scalia GM et al. Ejection fraction and mortality: a nationwide register-based cohort study of 499,153 women and men. Eur. J. Heart Fail 2021;23:406–416 [16]. Copyright © The Author(s) 2020. European Journal of Heart Failure published by John Wiley & Sons Ltd on behalf of European Society of Cardiology.

The findings of another study were similar, in which an increased risk for cardiovascular-related mortality persisted to a LVEF level of 60.0–64.9% in females, whereas in males the equivalent LVEF level was lower (55.0–59.9%) (Figure 2) [16].

Differences related to sex were also reported in the COronary CT Angiography EvaluatioN For Clinical Outcomes: An InteRnational Multicenter (CONFIRM) registry in whom LVEF was measured by cardiac computed tomography. Females with high LVEF died more often from any cause as compared to females with normal LVEF, while an opposite trend was observed in males [17]. Thus, the LVEF based terminology for HF classification is unjustified, especially at the higher end of the HF spectrum, as a LVEF > 60% cannot be considered normal or preserved based on recent evidence.

## 3. Positive Findings of Trials with Neurohormonal Inhibitors in HFpEF

Several trials testing the effectiveness of renin-angiotensin-aldosterone system (RAAS) inhibition in HFpEF have reported positive results.

Candesartan effectiveness was tested in the Candesartan in Heart Failure-Assessment of Reduction in Mortality and Morbidity (CHARM) Programme, including patients with HFmrEF (LVEF 40–49%, *n* = 1322), HFrEF (LVEF < 40%, *n* = 4323), and HFpEF (LVEF ≥ 50%, *n* = 1953) [18]. With LVEF as a continuous spline variable, candesartan significantly improved the primary outcome (cardiovascular death or HF hospitalization) until LVEF over 50% and recurrent HF hospitalizations until LVEF over 60%. It should be noted that in the I-PRESERVE trial, which demonstrated lack of benefit with irbesartan in HFpEF, approximately 25% of participants were treated with a combination of irbesartan and an angiotensin converting enzyme inhibitor (ACEi), a combination which has been abandoned due to complications [11].

The Treatment of Preserved Cardiac Function Heart Failure With an Aldosterone Antagonist (TOPCAT) trial randomized patients with symptomatic HF and a LVEF ≥ 45% (91% of participants suffered from HTN and none from VHD or HCM) to treatment with spironolactone or placebo [19]. Spironolactone did not significantly reduce the incidence of the primary composite outcome (a composite of death from cardiovascular causes, aborted cardiac arrest, or hospitalization for HF). However, a post hoc analysis demonstrated clinical benefits with spironolactone in HFpEF patients from the Americas than Russia or Georgia [20]. Further, canrenone (an active spironolactone metabolite) was undetectable in significantly more participants from Russia than the United States and Canada (30% vs. 3%, *p* < 0.001) [21]. As a result, the Food and Drug Administration (FDA) Cardiovascular and Renal Drugs Advisory Committee convened 7 years after the initial presentation of results to review the TOPCAT study [22] and issued an indication of spironolactone for the reduction of HF hospitalizations in patients with HF and LVEF ranging from 40% to 57%.

In the Prospective Comparison of ARNI with ARB Global Outcomes in HF With Preserved Ejection Fraction (PARAGON), which enrolled patients with New York Heart Association (NYHA) class II -IV HF, LVEF ≥ 45%, elevated level of natriuretic peptides, and structural heart disease (approximately 95% suffered from HTN and none form VHD or HCM), patients were randomized to receive sacubitril/valsartan or valsartan [23]. Sacubitril/valsartan did not result in a decrease in the primary outcome (a composite of total hospitalizations for HF and death from cardiovascular causes). However, when the data from Angiotensin Neprilysin Inhibition vs. Enalapril Heart Failure trial (PARADIGM, sacubitril/valsartan in patients with class II-IV HF and LVEF ≤ 40%) and PARAGON were combined, the therapeutic effects of sacubitril/valsartan vs. valsartan, varied by LVEF with treatment benefits, especially for HF hospitalization, extending to patients with HF and a LVEF up to 57% [24]. The PARAGON-HF and PARADIGM-HF trials demonstrated that the efficacy of sacubitril/valsartan vs. renin-angiotensin inhibition, on top of beta-blockers and mineralocorticoid receptor antagonists (MRA), varies with LVEF. Greater treatment benefit is observed in patients with low LVEF compared with higher LVEF, but effectiveness, particularly for HF hospitalization, remains measurable up until LVEF of approximately 60%. These findings are in accordance with the continuous spectrum theory of HF, which proposes that the deleterious neurohormonal overactivity and consequently the effectiveness of neurohormonal modulating agents progressively decline as LVEF increases, but both remain measurable at higher LVEF [25,26,27]. As a result, the FDA expanded the indication use of sacubitril/valsartan to include the HFpEF patient population based on the results of the PARAGON-HF trial.

Β-Blockers, which have been associated with favorable outcomes in chronic HF up to a LVEF of approximately 50%, have not been adequately investigated in randomized control trials in HfpEF in sinus rhythm [28]. Nevertheless, treatment with β-blockers is recommended in HfpEF complicated by HTN, atrial fibrillation (rate control), and coronary artery disease, which are present in most HfpEF patients. Thus, it is not surprising that in the PARAGON-HF trial approximately 80% of the patients were treated with concomitant β-blockers.

## 4. Positive Trials with SGLT2 Inhibitors in HfpEF

Sodium-glucose cotransporter 2 inhibitors (SGLT2i) have demonstrated a class effect in reducing HF hospitalizations in patients with or without baseline cardiovascular disease but also in preserving renal function [29,30]. The Dapagliflozin and Prevention of Adverse Outcomes in Heart Failure (DAPA-HF) trial, in which dapagliflozin was used [31], and the Empagliflozin Outcome Trial in Patients with Chronic Heart Failure and a Reduced Ejection Fraction (EMPEROR-Reduced), in which empagliflozin was used, proved beneficial in patients with HFrEF [32].

In the Empagliflozin Outcome Trial in Patients With Chronic Heart Failure With Preserved Ejection Fraction (EMPEROR-Preserved) trial, HfpEF patients with or without T2D (more than 90% with HTN and none with VHD or HCM) were randomized to empagliflozin or placebo [33]. Treatment with empagliflozin was associated with a 21% lower relative risk of the primary outcome (combined occurrence of cardiovascular death or hospitalization for HF (HHF)), predominantly due to the reduced number of HHF (11.8% in the placebo group vs. 8.6% in patients treated with empagliflozin). On the other hand, the study demonstrated a beneficial effect on renal function as patients receiving empagliflozin had a slower decline in eGFR when compared with placebo (1.25 mL per year decline in eGFR in the empagliflozin group vs. 2.62 mL per year decline in eGFR in placebo). As a result, the U.S. Food and Drug Administration approved empagliflozin to reduce the risk of cardiovascular death and hospitalization for HF in adults regardless of the LVEF.

**Figure 3:**
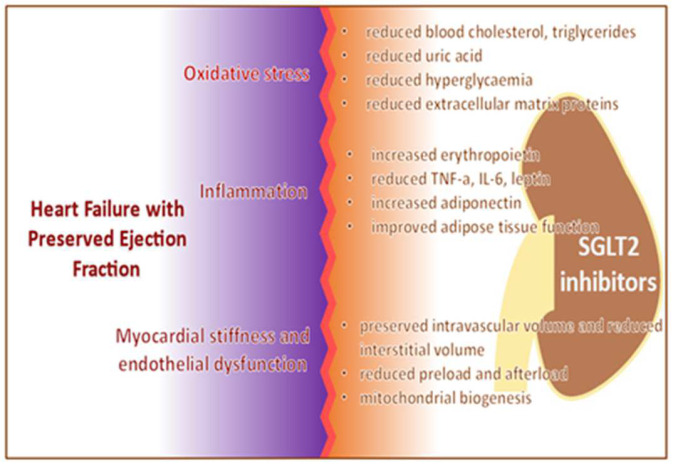
Mechanism of action of sodium glucose cotransporter 2 (SGLT2) inhibitors in heart failure with preserved ejection fraction.

The proposed cardioprotective mechanisms of SGLT2 inhibition (Figure 3) include diuresis and natriuresis, a decline in arterial blood pressure, augmented erythropoiesis, enhanced heart energy metabolism, decline in inflammation, inhibition of the sympathetic nervous system, reduction in oxidative stress, and improved endothelial function, among others [34].

## 5. Positive Trials Targeting Left Ventricular Filling Pressures in HFpEF

Tissue congestion caused by high cardiac filling pressures plays a crucial role in the pathogenesis of HFpEF, and interventions to reduce filling pressures improved outcomes in this patient population. In the CardioMEMS Heart Sensor Allows Monitoring of Pressure to Improve Outcomes in NYHA Class III Heart Failure Patients (CHAMPION) trial, clinical management guided by physician knowledge of central hemodynamics significantly reduced HF hospitalizations [35]. Similar were the findings in an ancillary analysis restricted to HFpEF [36] and in more recent analyses of Medicare beneficiaries [37]. In addition to direct hemodynamic monitoring, other methods to detect congestion allowing for intervention have proved to be promising, including the measurement of blood and plasma volume by radiolabeled indicator-dilution techniques [38].

## 6. The Contribution of Hypertension to HFpEF

The associations between comorbidities and the cardiovascular system as well as between comorbidities themselves are complex and may lead to the development of HF. Additionally, the other way round, HF per se is a “generator” of comorbidities adversely affecting prognosis [39]. Thus, prevention of comorbidities related to HF in the community (i.e., obesity and diabetes) is of utmost importance.

HTN is not a simple comorbidity but a major HF risk factor. Blood pressure management not only prevents asymptomatic HTN-mediated cardiac damage that can progress to HF, but can also prevent further disease deterioration [40]. Thus, although HFpEF patients suffer from several comorbidities, HTN is undoubtedly the most important [10]. This was demonstrated in a study including 1064 HF patients, in which there were 90 (8.5%) HF patients with a single coexisting morbidity, 33 (36.7%) with LVEF ≥ 50%, 27 (30.0%) with LVEF = 40–49%, and 30 (33.3%) with LVEF < 40%. In this single comorbidity subgroup, those with LVEF ≥ 50% suffered mostly from HTN (85.7%), whereas the second most common coexisting morbidity was atrial fibrillation, which is often a complication of HΤΝ (9.5%) [41]. These findings indicate that among HFpEF comorbidities the only one that can cause HFpEF by itself is HTN.

HTN in elderly HFpEF patients results from different pathophysiological mechanisms compared with middle aged or younger patients [42]. The predominant increase in the systolic arterial pressure observed in this patient population is indicative of an increase in aortic stiffness (a decrease in arterial compliance). Arterial load and stiffness are closely related to LV diastolic function, LV mass, and myocardial deformation [43]. In fact, LV diastolic dysfunction, LV hypertrophy, abnormal myocardial deformation, and increased aortic stiffness share many epidemiological and pathophysiological features as they predominate in elderly individuals and hypertensives, have a predictive outcome value, and share underlying mechanisms of remodeling including collagen deposition, increased cellular stiffness, and production of advanced glycation end-products in diabetes mellitus [43]. Furthermore, impairment of the coronary flow reserve is associated with increased arterial stiffness and myocardial dysfunction in HTN, inflammatory diseases, and coronary artery disease [44].

**Figure 4:**
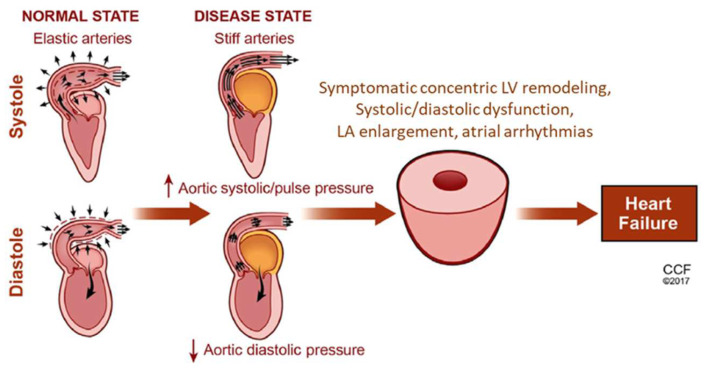
Effects of increased aortic stiffness on LV function and structure. LV, left ventricle; LA, left atrium; HF, heart failure. Reprinted with permission from Xanthopoulos A, Triposkiadis F and Starling RC. Heart failure with preserved ejection fraction: Classification based upon phenotype is essential for diagnosis and treatment. Trends Cardiovasc Med. 2018;28:392–400 [45]. Copyright © The Author(s) 2018. Trends in Cardiovascular Medicine published by Elsevier.

The increased aortic stiffness leads to increased velocity of the incident wave and an early return of the reflected wave and shifts pressure augmentation from diastole to systole. Systolic pressure and LV afterload rise leads to LV hypertrophy and increased myocardial oxygen demand, whereas aortic diastolic pressure (a major determinant of coronary perfusion pressure) drops, resulting in myocardial ischemia due to perfusion-metabolism mismatch (Figure 4) [45,46]. Currently there is no distinct intervention that reduces aortic stiffness, and the only effective treatment is the reduction of blood pressure, which is reduced both by RAAS and SGLT2 inhibitors. However, there is convincing evidence that intense blood pressure lowering (i.e., systolic blood pressure < 120 mmHg) is associated with a significantly lower incidence of major cardiovascular events (MACE), cardiovascular deaths, and all-cause death compared with the less intense blood lowering strategy (i.e., SBP ≥ 120 mmHg) [47].

Obesity has been associated with increased risk of HF development, irrespective of the LVEF. Patterns of LV remodeling in obese patients include concentric LV remodeling, concentric LV hypertrophy, and eccentric LV hypertrophy. The underlying mechanisms include hemodynamic and neurohormonal factors, the effect of coexisting morbidities and inflammation [48]. The “obesity paradox” has been a long-standing controversy, with many studies demonstrating a protective effect of obesity in patients with HF. Potential explanations include reverse epidemiology, the presence of anti-inflammatory adipokines and earlier appearance of symptoms in obese individuals, and the limitations of body mass index (BMI) as a measure of obesity. In this regard, a novel simple index, the waist-corrected BMI, calculated as waist circumference × BMI, has been proposed. This index considers both the global fat mass and distribution and might be useful for a better cardiovascular risk assessment [49].

## 7. HFpEF Treatment in Clinical Practice

**Figure 5:**
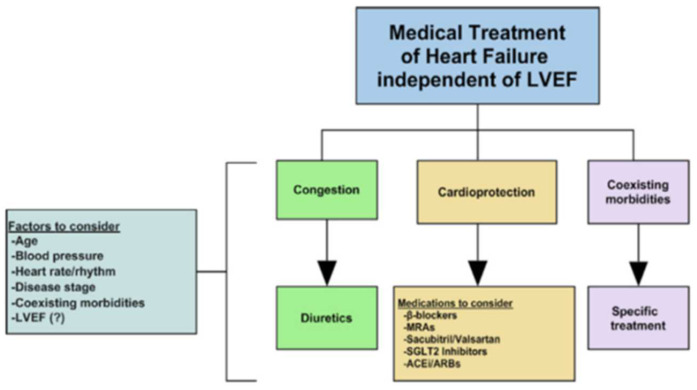
Medical treatment of heart failure (HF) irrespective of left ventricular ejection fraction (LVEF). SGLT2, sodium/glucose cotransporter 2; MRAs, mineralocorticoid receptor antagonists; ACEi, angiotensin-converting enzyme inhibitors; ARBs, angiotensin receptor blockers. Reprinted with permission from Triposkiadis F, Xanthopoulos A and Starling RC. Medical Treatment of Heart Failure: Ignore the Ejection Fraction and Treat All? J. Card Fail. 2021;27:907–909 [50]. Copyright © The Author(s) 2021. Journal of Cardiac Failure published by Elsevier.

Treatment of HfpEF has been considered an unmet need for years. There are five main reasons in our opinion. (a) The ever-changing LVEF cut offs used for HF classification; (b) controversies regarding the definition of the LVEF normal range; (c) the fact that HFpEF does not represent a phenotype, but a category of diseases with entirely different characteristics (hypertensive heart disease, VHD, HCM etc.); (d) the lack of recognition that hypertensive HFpEF is the most common and important HFpEF phenotype; (e) the assumption that neurohormonal overactivity is absent in HF patients with LVEF > 45–50%, which has been proved wrong. The findings of recent studies indicate that the four major classes of medications used in HF—β-blockers, mineralocorticoid receptor antagonists, sacubitril-valsartan /ACEi/angiotensin receptor blockers (ARB), and SGLT2 inhibitors—are effective not only in HFrEF, but in HF including a wider range of LVEFs and especially in hypertensive HFpEF (Figure 5) [50]. Endorsed by the 2022 AHA/ACC/HFSA Guideline for the Management of Heart Failure, SGLT2 inhibitors now have a Class 2a Recommendation in the treatment algorithm of HFpEF and HFmrEF, whereas MRAs and ARNi have a 2b Class Recommendation [10]. Thus, restricting treatment with these agents to HF patients with LVEF < 40% is not supported by the current evidence. The sine qua non to decide whether to treat should be based on the diagnosis of HF, which typically includes congestion and elevation of natriuretic peptides regardless of the LVEF. Additional aggressive treatment of coexisting morbidities is mandatory.

## 8. Conclusions

Treatment of HFpEF, especially in hypertensive HFpEF which is the most common, should not be considered an unmet need anymore. Based on the contemporary evidence, the role of LVEF in the HF classification is doubtful. In this regard, a HF classification based on causes or risk factors would be the most reasonable approach. Despite the greater effectiveness of neurohormonal inhibitors in the lower end (vs. the higher end) of HF spectrum, restricting the four major classes of medications used for HF treatment in patients with a LVEF < 40% is not justified anymore.

## Data Availability

Not applicable.
